# Microsatellite Instability Occurs Rarely in Patients with Cholangiocarcinoma: A Retrospective Study from a German Tertiary Care Hospital

**DOI:** 10.3390/ijms19051421

**Published:** 2018-05-09

**Authors:** Ria Winkelmann, Markus Schneider, Sylvia Hartmann, Andreas A. Schnitzbauer, Stefan Zeuzem, Jan Peveling-Oberhag, Martin Leo Hansmann, Dirk Walter

**Affiliations:** 1 Senckenberg Institute of Pathology, Johann Wolfgang Goethe-University Hospital, Theodor-Stern-Kai 7, 60590 Frankfurt, Germany; Winkelmann@em.uni-frankfurt.de (R.W.); s.hartmann@em.uni-frankfurt.de (S.H.); m.l.hansmann@em.uni-frankfurt.de (M.L.H.); Dirk.Walter@kgu.de (D.W.); 2 Department of General and Visceral Surgery, Johann Wolfgang Goethe-University Hospital, Theodor-Stern-Kai 7, 60590 Frankfurt, Germany; AndreasAnton.Schnitzbauer@kgu.de; 3 Department of Internal Medicine I, Johann Wolfgang Goethe-University Hospital, Theodor-Stern-Kai 7, 60590 Frankfurt, Germany; Stefan.Zeuzem@kgu.de (S.Z.); jan.peveling-oberhag@rbk.de (J.P.-O.); 4 Department for Gastroenterology, Hepatology and Endocrinology, Robert-Bosch Hospital, Auerbachstraße 110, 70376 Stuttgart, Germany

**Keywords:** microsatellite instability, cholangiocarcinoma, immunohistochemistry, PCR

## Abstract

Immune-modulating therapy is a promising therapy for patients with cholangiocarcinoma (CCA). Microsatellite instability (MSI) might be a favorable predictor for treatment response, but comprehensive data on the prevalence of MSI in CCA are missing. The aim of the current study was to determine the prevalence of MSI in a German tertiary care hospital. Formalin-fixed paraffin-embedded tissue samples, obtained in the study period from 2007 to 2015 from patients with CCA undergoing surgical resection with curative intention at Johann Wolfgang Goethe University hospital, were examined. All samples were investigated immunohistochemically for the presence of MSI (expression of MLH1, PMS2, MSH2, and MSH6) as well as by pentaplex polymerase chain reaction for five quasimonomorphic mononucleotide repeats (*BAT-25*, *BAT-26*, *NR-21*, *NR-22*, and *NR-24*). In total, 102 patients were included, presenting intrahepatic (*n* = 35, 34.3%), perihilar (*n* = 42, 41.2%), and distal CCA (*n* = 25, 24.5%). In the immunohistochemical analysis, no loss of expression of DNA repair enzymes was observed. In the PCR-based analysis, one out of 102 patients was found to be MSI-high and one out of 102 was found to be MSI-low. Thus, MSI seems to appear rarely in CCA in Germany. This should be considered when planning immune-modulating therapy trials for patients with CCA.

## 1. Introduction

Cholangiocarcinoma (CCA) is a gastrointestinal neoplasia which derives from the biliary epithelium or peribiliary glands within the biliary tree. It is subdivided into intrahepatic (iCCA) and extrahepatic perihilar (pCCA) and distal cholangiocarcinoma (dCCA). The five-year survival rates of patients with CCA remain below 20% despite surgery, and no targeted therapy regimen has demonstrated a therapeutic benefit compared to the standard therapy of gemcitabine and cisplatin, so far [1,2].

In recent years, a new promising approach in cancer therapy has evolved with the emergence of immune-modulating monoclonal antibodies. These agents focus on receptors or ligands of effector cells as targets for cancer immunotherapy by inhibiting immune check points such as the programmed cell death protein 1 (PD-1, CD279) and its ligand (PD-L1) or the protein receptor CTLA4 (CD152). Objective responses in patients treated with anti-PD1 antibodies were seen in patients with advanced unresectable melanoma and non-small-cell lung cancer or undergoing second-line therapy for metastatic renal-cell carcinoma [3,4,5,6]. Moreover, promising results have been shown for various other solid tumors and hematologic malignancies [7].

Notably, the treatment with an immune-modulating therapy, especially when agents are combined, may lead to severe side effects. Furthermore, only a certain percentage of patients seems to respond to the treatment, which highlights the importance to develop predictive biomarkers. Besides the immunohistochemical evaluation of PD-L1 expression, microsatellite instability (MSI) seems to be associated with an improved clinical response rate: MSI induces somatic hypermutation and consecutively neoepitopes, which might create an immune response [8].

According to recent data, the immune-modulating therapy might be a promising alternative to standard treatments for MSI-positive CCA [9]. Moreover, a durable response to immune checkpoint inhibition was shown in a patient with an advanced-stage, microsatellite-unstable CCA [10]. However, as shown in a recent review, data on MSI in CCA is very limited [11]. The study sizes were small, or only certain groups of CCA patients, such as those affected by liver fluke-induced iCCA, which occurs rarely in western countries, were investigated. Given the devastating prognosis currently faced by patients with advanced CCA, further characterization of the group of patients potentially profiting from immune-modulating therapy regimens is urgently warranted.

The aim of the current study was to determine the presence of MSI in all subclasses of CCA by immunohistochemistry and polymerase chain reaction (PCR) in a retrospective analysis at a German tertiary care hospital.

## 2. Results

### 2.1. Clinicopathological Characteristics

**Figure 1:**
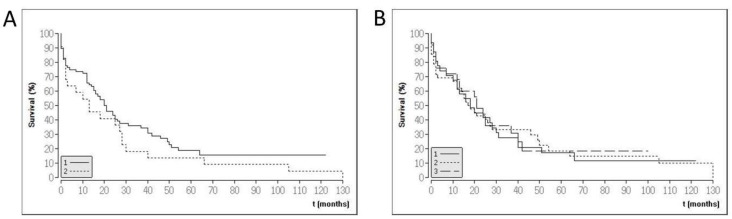
Kaplan–Meier graphs of the study population. (**A**) Survival data for moderate (group 1: UICC stadium I and II) and advanced (UICC stadium III and IV) cholangiocarcinoma (CCA) patients. (**B**) Survival data according to CCA location in the biliary tree (1: intrahepatic CCA, 2: perihilar CCA, 3: distal CCA).

In total, 102 patients (71 male, 70%) were included. The cohort consisted of intrahepatic (*n* = 35; 34.3%) and extrahepatic CCA (perihilar: *n* = 42, 41.2%; distal: *n* = 25, 24.5%). The majority of the included patients had a moderate tumor stage (Union internationale contre le cancer (UICC) I and II) at the time of resection. The mean age at diagnosis was 66 (range 38–84 years, standard deviation (SD) 10.9). In total, 87 out of 102 patients (85%) had died by the study closure (median survival 16 months, range 0–130 months, SD 25.5). For two patients, no follow-up data were available. Of the remaining thirteen patients who were alive, the median follow up was 37 months (range 18–122 months, SD 31.6). The clinicopathological characteristics are provided in Table 1. Kaplan–Meier graphs are provided in Figure 1.

### 2.2. Immunohistochemistry

**Figure 2:**
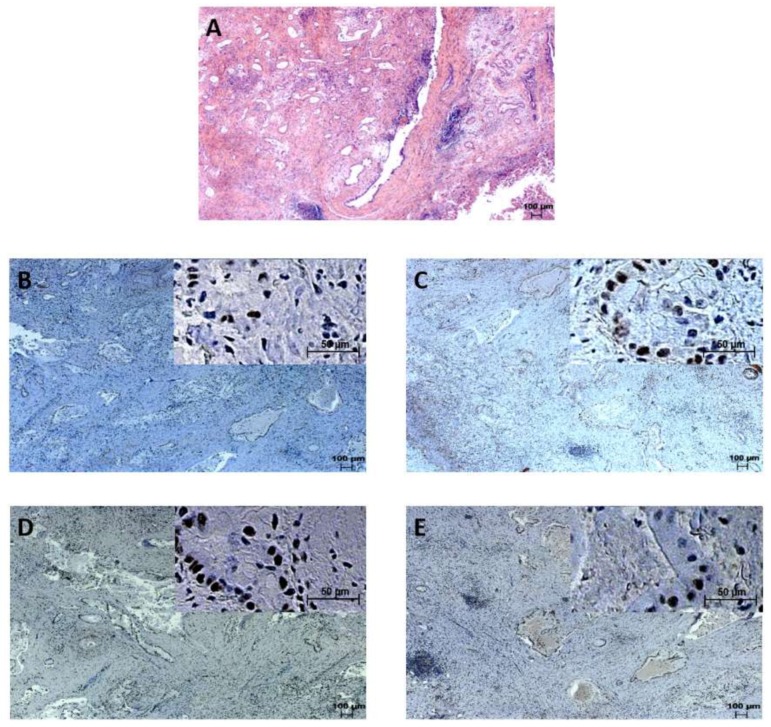
Extrahepatic cholangiocarcinoma staining. (**A**): Hematoxylin and eosin (HE) staining. Samples immunohistochemically positive for (**B**): PMS2, (**C**): MLH1, (**D**): MSH6, (**E**): MSH2.

MSI was immunohistochemically evaluated by analyzing the expression of MLH1, PMS2, MSH2, and MSH6. All 102 patients were positive for MLH1, PMS2, MSH2, and MSH6. Thus, no MSI was detected via immunohistochemistry. Representative examples of the expression of mismatch repair proteins are shown in Figure 2.

### 2.3. Microsatellite Instability Analysis via PCR

**Figure 3:**
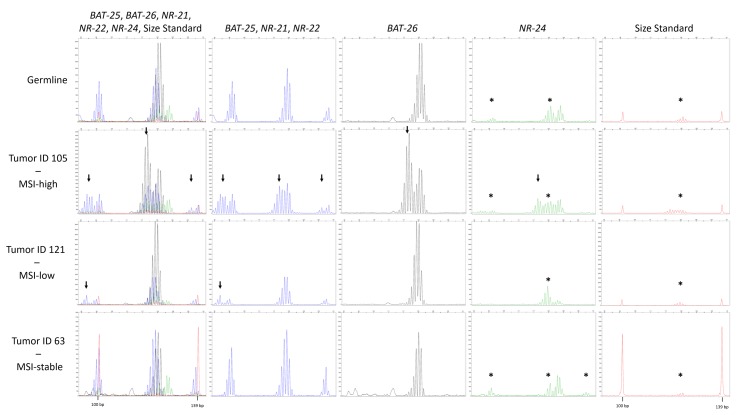
Typical allelic profiles of BAT-25, BAT-26, NR-21, NR-22, and NR-24. Only the range from 92 to 142 bp of the electropherograms comprising the region of interest is shown. The red line indicates the GeneScan 350 ROX^TM^ dye Size Standard labeled with ROX displaying the 100 bp and 139 bp peak. The first column shows all traces, the second column shows the fluorescein (FAM)-labeled products (*BAT-25*, *NR-21*, and *NR-22*) depicted in blue. In the third column, the ATTO55O-labeled *BAT-26* is separately displayed in black. HEX-labeled *NR-24* is shown separately in green in column 4, and the fifth column shows the size standard in red. The expected sizes for the products *NR-21*, *BAT-25*, *BAT-26*, *NR-24*, and *NR-22* were 99, 123, 124, 128, and 139 bp, respectively. The top panel shows the normal germline alleles with typical stutter bands. In the lower panels, the electropherograms of the only microsatellite instability (MSI)-high tumor sample (ID 105), the only MSI-low tumor sample (ID 121), and one MSI-stable tumor sample (ID 63) is shown. The arrows indicate the presence of shifted alleles. The asterisk depicts artifacts coming from bleed-trough from the FAM signal into the other signals, identifiable by peaks in the other colors occurring at the exact same size as the FAM peaks.

**Figure 4:**
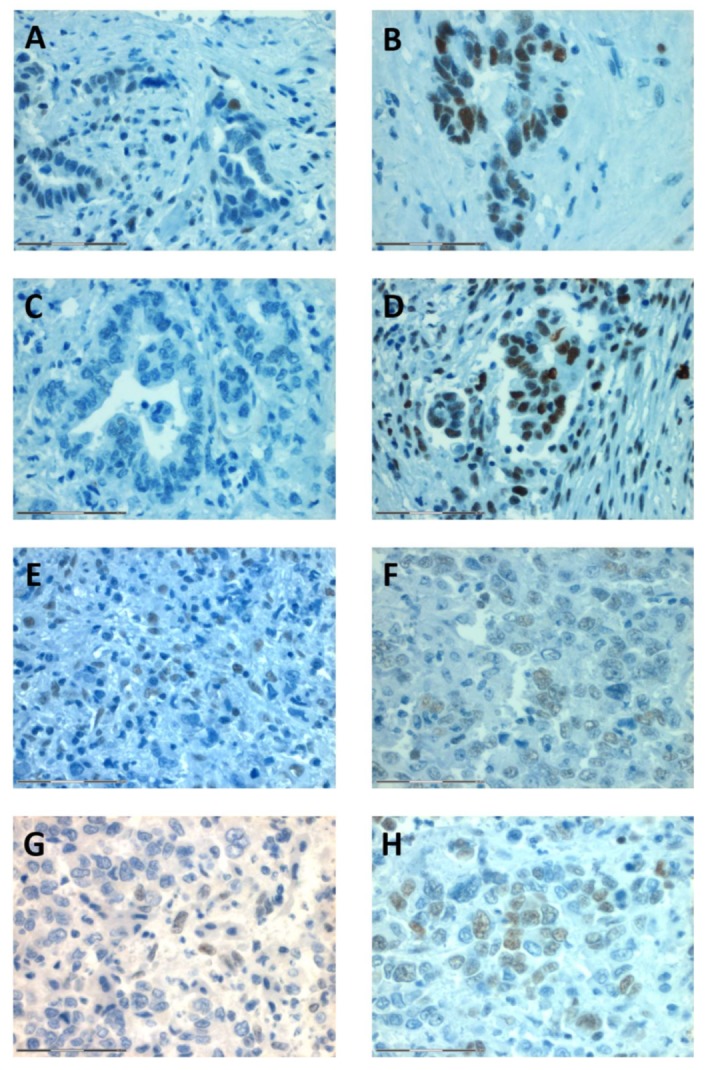
Representative stainings of the patients with MSI-low CCA (Pat ID 121, **A**–**D**) and MSI-high CCA (Pat ID 105, **F**–**H**). In both patients, no loss of expression was found for PMS2 (**A**,**E**), MLH2 (**B**,**F**), MLH1 (**C**,**G**), and MSH6 (**D**,**H**). Magnification: 40×, scale bar: 75 μm.

Besides immunohistochemistry, MSI was evaluated using five quasimonomorphic mononucleotide repeats (*BAT-25*, *BAT-26*, *NR-21*, *NR-22*, and *NR-24*) in a pentaplex PCR, as described in Suraweera et al. 2002 [12]. Thereby, one case (Pat ID 121) was found to be MSI-low with alterations in locus *NR-21*, and one case (Pat ID 105) was diagnosed as MSI-high, since all analyzed MSI biomarkers were found to be unstable (Figure 3). However, no loss of expression of a DNA repair enzyme was found immunohistochemically in these two cases (Figure 4).

Patient 121 (male, age at diagnosis 49 years) and patient 105 (female, age at diagnosis 75 years) had pCCA.

## 3. Discussion

The prognosis of patients with advanced CCA is poor, and new treatment options are warranted. Recent data suggested that patients with microsatellite-unstable CCA might profit from immune-modulating therapy. In the current study, we provide data on prevalence of MSI in the so far largest cohort of western patients including all subtypes of CCA. We thereby observed a very low prevalence of MSI, with 1% of MSI-high and 1% of MSI-low tumors.

Because of the emerging role of MSI in personalized cancer therapy, MSI prevalence was increasingly investigated in recent years. In a study based on exome data of The Cancer Genome Atlas (TCGA), MSI was analyzed in 18 cancer types, and MSI-positive tumors were found in 14 of 18 entities [13]. The proportion of MSI positivity ranged from nearly 30% in uterine corpus endometrial carcinoma, to about 20% in colon adenocarcinoma and stomach adenocarcinoma, to below 5% in kidney renal clear cell carcinoma, rectal adenocarcinoma, prostate adenocarcinoma, ovarian serous cystadenocarcinoma, glioblastoma multiforme lung adenocarcinoma, head and neck squamous cell carcinoma, hepatocellular carcinoma (HCC), lung squamous cell carcinoma, bladder urothelial carcinoma, and lower grade brain glioma. No MSI was observed in breast invasive carcinoma, skin cutaneous melanoma, kidney renal papillary cell carcinoma, and thyroid carcinoma. Two of 338 HCC (0.6%) were MSI-high, which, combined with the data of the present study, indicates a rare occurrence of MSI in hepatobiliary tract cancer. This should be kept in mind when considering implementing MSI testing in diagnostic routines to select patients potentially profiting from immune-modulating therapy.

The low positivity of MSI in CCA revealed in our study seems consistent with earlier data. For example, a study including 37 patients with liver fluke-induced iCCA as well as a study including 38 patients with extrahepatic CCA (eCCA) observed no patient (0%) with MSI-high tumors [14,15]. Notably, some studies found higher rates of MSI-high tumors as well. For instance, MSI-high iCCA were found in 14% and 18% in two studies including 22 patients each [16,17]. Likewise, a study including 28 patients with eCCA from 2002 found two patients (7%) to be MSI-high [18]. Of note, all these studies included Asian patients. Since the etiology might vary in different countries, a comparison of our study and former data is impeded. Because of the retrospective structure of the current study, ethnicity and country of origin could not be assessed. Despite that, the majority of patients usually treated at University hospital Frankfurt consists of Caucasians, and we hypothesize that the results of the current study indicate a very low prevalence of MSI in CCA in western countries.

Besides the geographical differences, the methodological approach of different studies should be considered as well when comparing different studies investigating MSI. In the present study, we used the commonly used detection of MSI via immunohistochemistry (MLH1, PMS2, MSH2, and MSH6) and, in addition, a well-tested method including five quasimonomorphic mononucleotide repeats performing equally to the Bethesda panel in terms of sensitivity and specificity in the detection of MSI in colorectal cancer [12]. Discordant results of immunohistochemistry and PCR-based techniques were observed in this study. Notably, it has been described that in some cases MSI can be detected via a PCR approach without concurrent loss of expression of any of the four mismatch repair proteins, as these have lost their function as a result of mutations [19,20,21]. Thus, discrepancies between the two methods have been described for other entities such as colorectal cancer, indicating to view these techniques as complimentary. We therefore recommend using both methods to investigate the MSI status in CCA in future studies. In other studies on CCA, the number of the MSI markers evaluated by PCR varied between five and more than 10 [15,16]. Besides, Hause et al. included over 200,000 loci and used an exome-based classifier to identify MSI-high tumors [13]. By analyzing MSI on a whole-exome basis, they observed a variation of microsatellite instable loci across cancer types, indicating that loci being inherently stable in one cancer type might be frequently mutated in another. On the basis of these observations, a comprehensive whole exome-based analysis of MSI in cholangiocarcinoma is of great interest.

In conclusion, the data of the current study indicate that MSI occurs rarely in western CCA patients. This should be kept in mind in the planning of future therapy trials including patients with MSI-high CCA.

## 4. Materials and Methods

### 4.1. Patients

Formalin-fixed, paraffin-embedded tissue samples from patients with CCA undergoing surgical resection in the period from 2007 to 2015 were obtained from the archive of the Senckenberg Institute of Pathology, University Hospital Frankfurt. The clinical data (date of birth, gender, tumor stage, tumor size, and follow-up data) were retrieved from electronic medical records. Only patients without prior treatment who underwent surgery with curative intention were included. Pathologic tumor-node-metastasis stages were assessed with the international system for staging biliary tract cancer adopted by the American Joint Committee on Cancer and the UICC (Union Internationale Contre le Cancer), 7th edition [22]. The clinical data and biomaterial were obtained from the tumor documentation and the biobank of the Universitäre Centrum für Tumorerkrankungen (UCT) Frankfurt (University Cancer Center, Frankfurt, Germany). Written informed consent was available from all patients. The study was approved by the institutional Review Boards of the UCT and the Ethics Committee at the University Hospital Frankfurt (Approval No. SGI-07-2016, date 23 February 2017).

### 4.2. Immunohistochemistry

Freshly cut paraffin sections, 4-μm-thick, were stained for MLH1, MSH6, PMS2, and MSH2. The antibodies used are described in Table 2. The immunohistochemical stainings were judged by two pathologists (Ria Winkelmann, Sylvia Hartmann) independently, and the assessment was performed without awareness of the MSI status of each case. CCA were staged as negative if none of the tumor cells were stained. In case of disagreement, the slides were reviewed again, and a consensus was reached. Lymphocytes, stromal cells, and non-neoplastic epithelium served as internal positive controls.

### 4.3. DNA Extraction from Formalin-Fixed, Paraffin-Embedded (FFPE) Tissue Samples

After macrodissection, DNA was extracted with the Maxwell 16 FFPE tissue LEV DNA purification kit (Promega, Madison, WI, USA) from FFPE material according to the manufacturer’s recommendations. DNA yield was quantified with Quantus Fluorometer (Promega).

### 4.4. PCR and Microsatellite Instability (MSI) Analysis

MSI analysis was adapted from Suraweera et al. 2002 [12]. In brief, five quasimonomorphic monocleotide markers (*BAT-25*, *BAT-26*, *NR-21*, *NR-22*, and *NR-24*) were amplified in a single multiplex PCR reaction. The primer sequences and fluorescence labeling of one primer of each primer pair is given in Table 3. PCR was performed with the Taq PCR Mastermix from Qiagen (Hilden, Germany), according to the manufacturer’s directions. All primers were used at a final concentration of 240 pmol/L, except for the primers for *BAT-25*, which were used at the concentration of 1 μmol/L. Ten to 50 ng of DNA was used as input material. The following PCR conditions were applied: denaturation at 95 °C for 5 min, 35 cycles of denaturation for 30 s, annealing at 55 °C for 30 s and extension at 72 °C for 30 s, and a final extension at 72 °C for 10 min. The PCR products were analyzed on a 3130xl Genetic Analyzer (ThermoFisher Scientific, Darmstadt, Germany) with the 3130 Series Data Collection Software v.3.0. A 50 cm capillary array and the Fragment Analysis 50_POP7 settings were used. For size estimation, the GeneScan 350 ROX^TM^ dye Size Standard (ThermoFisher Scientific) was added to each sample. The final evaluation of the fragment length was done with the GeneMapper Software 5.0 (ThermoFisher Scientific). A sample was considered MSI-high or MSI-low if more than three or one–two markers represented shifted alleles, respectively.

### 4.5. Statistics

Descriptive statistics, such as the calculation of mean value, range, and standard deviation, as well as the calculation of Kaplan–Meier graphs, were determined using BiAS (version 11.01, BiAS for Windows; Epsilon-Verlag, Frankfurt, Germany). The overall survival was calculated as time from surgery to date of death (event) or date of the last follow-up (censored).

## Figures and Tables

**Table 1:** Clinicopathological characteristics of the patient cohort.

Variable	Variable	N	%
Sex	Male	71	69.6
	Female	31	30.4
Localization	iCCA	35	34.3
	pCCA	42	41.2
	dCCA	25	24.5
Age	≥65	61	59.8
	<65	41	40.2
UICC	1	35	34.3
	2	43	42.2
	3	16	15.7
	4	8	7.8
T	1	20	19.6
	2	50	49.0
	3	29	28.4
	4	3	2.9
N *	0	61	59.8
	1	40	39.2
L *	0	56	54.9
	1	37	36.3
V *	0	14	13.7
	1	79	77.5
Pn *	0	66	64.7
	1	25	24.5
R	0	76	74.5
	1	26	25.5
G	1	3	2.9
	2	77	75.5
	3	22	21.6

iCCA: intrahepatic cholangiocarcinoma, pCCA: perihilar cholangiocarcinoma, dCCA: distal cholangiocarcinoma (dCCA), UICC staging according to the 7th edition. TNM classification: T: extent of the primary tumor, N: spread to regional lymph nodes, L: invasion into lymphatic vessels, V: invasion into veins, Pn: perineural invasion, R: resection boundaries free of cancer or not, G: grading; * data were not available for all patients.

**Table 2:** Antibodies.

Antibody	Supplier	Clone	Dilution	Pretreatment
MLH1	BD Pharmingen^TM^ (Franklin Lakes, NJ, USA)	G168-728	1:750	Microwave 15’, EDTA, pH 8
MSH2	Calbiochem^®^ (Darmstadt, Germany)	GB12	1:50	Microwave 15’, EDTA, pH 8
MSH6	DCS (Hamburg, Germany)	SP93	RTU	Water bath, Trilogy 30’, pH 8
PMS2	BD Pharmingen^TM^	A16-4	1:40	Water bath 60’, pH 9

RTU: ready to use.

**Table 3:** Primer for MSI analysis.

Name	Fluorescent Marker	Sequence (5‘→3‘)	Expected PCR Product Size (bp)
NR-21_For	FAM	TAAATGTATGTCTCCCCTGG	99
NR-21_Rev		ATTCCTACTCCGCATTCACA	
BAT-26_For	ATTO0550	TGACTACTTTTGACTTCAGCC	24
BAT-26_Rev		AACCATTCAACATTTTTAACCC	
BAT-25_For	FAM	TCGCCTCCAAGAATGTAAGT	123
BAT-25_Rev		TCTGCATTTTAACTATGGCTC	
NR-24_For	HEX	CCATTGCTGAATTTTACCTC	128
NR-24_Rev		ATTGTGCCATTGCATTCCAA	
NR-22_For	FAM	GAGGCTTGTCAAGGACATAA	139
NR-22_Rev		AATTCTGATGCCATCCAGTT	

## Abbreviations

CCA	Cholangiocarcinoma
dCCA	Distal CCA
eCCA	Extrahepatic CCA
FAM	Fluorescein
FFPE	Formalin-Fixed, Paraffin-Embedded
HCC	Hepatocellular carcinoma
HE	Hematoxylin and eosin
iCCA	Intrahepatic CCA
MSI	Microsatellite instability
pCCA	Perihilar CCA
RTU	Ready to use
SD	Standard deviation
TCGA	The Cancer Genome Atlas
UCT	Universitäre Centrum für Tumorerkrankungen
UICC	Union internationale contre le cancer
